# Effects of Myristicin in Association with Chemotherapies on the Reversal of the Multidrug Resistance (MDR) Mechanism in Cancer

**DOI:** 10.3390/ph15101233

**Published:** 2022-10-07

**Authors:** Elisa Frederico Seneme, Daiane Carla dos Santos, Carolina Afonso de Lima, Ícaro Augusto Maccari Zelioli, Juliana Mozer Sciani, Giovanna Barbarini Longato

**Affiliations:** 1 Research Laboratory in Molecular Pharmacology of Bioactive Compounds, São Francisco University, Bragança Paulista 12916900, SP, Brazil; 2 Laboratory of Optimization, Design and Advanced Control (LOPCA), School of Chemical Engineering, University of Campinas (UNICAMP), Campinas 13083872, SP, Brazil; 3 Multidisciplinary Research Laboratory, São Francisco University, Bragança Paulista 12916900, SP, Brazil

**Keywords:** multidrug resistance, glycoprotein-P, myristicin, cisplatin, docetaxel

## Abstract

A range of drugs used in cancer treatment comes from natural sources. However, chemotherapy has been facing a major challenge related to multidrug resistance (MDR), a mechanism that results in a decrease in the intracellular concentration of chemotherapeutic agents, resulting in reduced treatment efficacy. The protein most frequently related to this effect is P-glycoprotein (P-gp), which is responsible for promoting drug efflux into the extracellular environment. Myristicin is a natural compound isolated from nutmeg and has antiproliferative activity, which has been reported in the literature. The present study aimed to evaluate the effect of the association between myristicin and chemotherapeutic agents on the NCI/ADR-RES ovarian tumor lineage that presents a phenotype of multidrug resistance by overexpression of P-gp. It was observed that myristicin showed no cytotoxic activity for this cell line, since its IC50 was >1 mM. When myristicin was associated with the chemotherapeutic agents cisplatin and docetaxel, it potentiated their cytotoxic effects, a result evidenced by the decrease in their IC50 of 32.88% and 75.46%, respectively. Studies conducted in silico indicated that myristicin is able to bind and block the main protein responsible for MDR, P-glycoprotein. In addition, the molecule fits five of the pharmacokinetic parameters established by Lipinski, indicating good membrane permeability and bioavailability. Our hypothesis is that, by blocking the extrusion of chemotherapeutic agents, it allows these agents to freely enter cells and perform their functions, stopping the cell cycle. Considering the great impasse in the chemotherapeutic treatment of cancer that is the MDR acquired by tumor cells, investigating effective targets to circumvent this resistance remains a major challenge that needs to be addressed. Therefore, this study encourages further investigation of myristicin as a potential reverser of MDR.

## 1. Introduction

Cancer is the main public health problem in the world. In most countries, it is the leading cause of death before age 70. The Global Cancer Observatory (GLOBOCAN) estimates that, in 2020 alone, there were 19.3 million new cases of cancer (18.1 million excluding non-melanoma skin cancer) and almost 10 million deaths due to the disease. The most common is breast cancer with an estimated 2.3 million new cases (11.7%), followed by lung (11.4%), colorectal (10.0%), prostate (7.3%) and stomach (5.6%) cancer. Studies indicate that, in 2040, there may be a 47% increase in cases compared to 2020, reaching 28.4 million cases [1].

Cancer treatment seeks to cure, prolong and improve the patient’s quality of life. Each type of cancer has a different clinical protocol, defined according to the location and tumor stage at diagnosis. The most frequently used therapy for the treatment of cancer is chemotherapy. The drugs used directly in the cell, interfere in processes of different phases of the cell cycle, and, therefore, they do not have great selectivity. Due to the low specificity, one of the main difficulties with this therapy is its ability to damage normal cells, causing high levels of toxicity to the patient’s organism and resulting in several side effects [2,3,4].

Despite the availability of several drugs of different pharmacological classes, there is a major barrier in chemotherapy treatment represented by multidrug resistance (MDR). MDR occurs when, after starting the use of anticancer drugs, the cancer cells acquire resistance mechanisms that cause a decrease in treatment effectiveness in 90% of cases. These mechanisms can be classified into two groups: the classical pathway and the non-classical pathway. The non-classical pathway is characterized by mechanisms related to cellular metabolism (GST proteins, topoisomerase, growth factors) that alter the mechanism of drugs or interfere with their effect. The classical pathway is related to decreased intracellular drug concentration. The drug reaches the intracellular environment through plasma membrane transport channels. Therefore, mutations that alter the activities of these proteins, or increase their expression, can cause an increased efflux of drug into the extracellular environment, reducing its effects [5].

The main group of transport proteins related to MDR is called the ABC family (from ATP-binding cassette protein). These proteins transport substances through the plasma membrane by the hydrolysis of ATP, reducing its concentration intracellularly. The main representative is called MDR1, also known as P-glycoprotein (P-gp), and its overexpression is related to the failure of treatments for different types of tumors [5].

Studies conducted with several substances already used in therapy, such as calcium channel blockers, immunosuppressants and antimalarials, showed toxicities and little significant reversal of the MDR. Several classes of natural compounds have been studied over the last few years and showed a positive response in the modulation of chemoresistance. The origin of such classes varies from plants to microorganisms and marine sources. The mechanisms responsible for the activity of most of these compounds are related to the ability to block P-gp and other membrane channels, in addition to reducing the expression of these proteins, resulting in a synergistic effect when associated with drug antineoplastics. The main classes that show such a positive effect are carotenoids (xanthines, lycopene, lutein), flavonoids (rutin, quercetin, chalcones, among others), alkaloids (indoles, steroids, piperidine derivatives, quinolines), cardiotonic steroids, coumarins, peptides and terpenoids. These studies show that, as in the general setting of therapy with medicinal products, natural products are a source of great importance in obtaining substances with the potential to reverse chemotherapy-resistant cancers [5,6]. For this reason, compounds of natural origin have been researched not only in the search for new antineoplastics but also to assess their MDR modulating activity [6].

Myristicin (1-allyl-3,4-methylenedioxy-5-methoxybenzene) is an active natural substance from the alkylbenzene family, mainly found in nutmeg (*Myristica fragrans*). It is also present in parsley (*Petroselinum crispum*), in black pepper (*Piper nigrum*), carrots (Umbelliferae family) and plants of the Apiaceae family. Historically, nutmeg has been used to treat illness such as cholera, diarrhea, stomach cramps, nausea and anxiety. It is believed that myristicin is the compound responsible for the benefits of nutmeg, since it is the main compound in this spice. Several studies have been conducted with this molecule in the last decades, demonstrating some biological activities with therapeutic potential. These studies show that the myristicin has anti-inflammatory and antioxidant properties, antimicrobial activity against pathogenic bacteria and fungi, insecticide and larvicide effects and also antiproliferative activity against several cancer cell lines [7].

**Figure 1:**
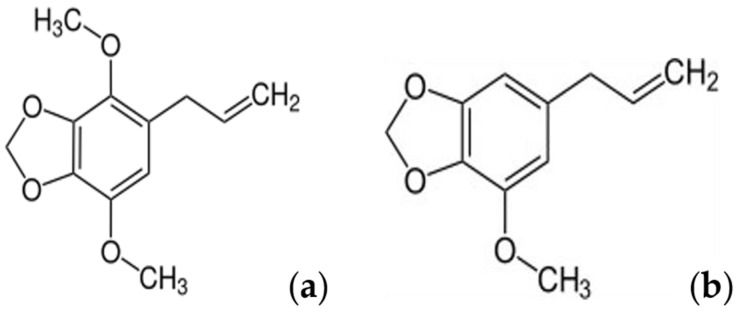
Molecular structure of the compounds apiole (**a**) and myristicin (**b**).

Although some studies have addressed the antiproliferative activity of myristicin, none of them investigated its potential mechanism of MDR reversal. Considering the similarity of the myristicin molecular structure with compound apiole recently studied by our research group (Figure 1) and whose results indicated it is a potential reverser of MDR [8], the aim of this research was to investigate the effects of myristicin in association with chemotherapeutic agents in a multidrug-resistant cell line, as well as its binding affinity for P-gp efflux pump.

## 2. Results

### 2.1. Association of Myristicin with Cisplatin and Docetaxel on NCI/ADR-RES Cell Line

The cytotoxic activity of the myristicin compound, as well as the chemotherapeutic agents cisplatin and docetaxel, was evaluated against the NCI-ADR/RES tumor line. From the curve obtained, IC50 values were calculated. The myristicin compound did not show cytotoxic activity, since its IC50 was >1 mM for this strain (Table 1). The chemotherapy drugs cisplatin and docetaxel, when administered alone, presented an IC50 of 215.60 ± 6.36 and 15.04 ± 1.36 μM, respectively (Table 1 and Table 2).

**Figure 2:**
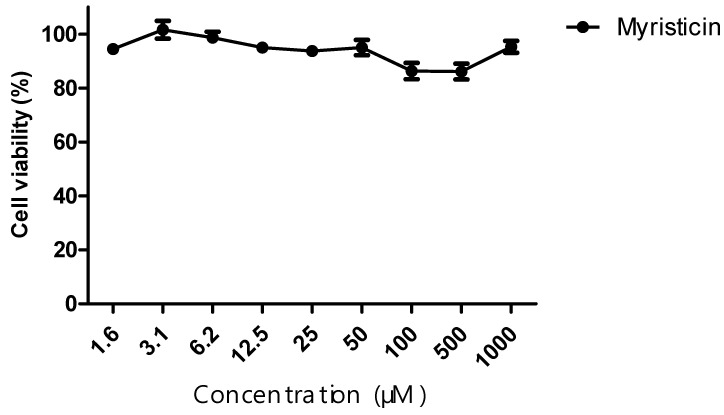
Cytotoxic activity of the myristicin compound. Assay performed with the NCI/ADR-RES tumor line, relating the percentage of cell viability versus concentration of myristicin, after 48 h of incubation.

**Figure 3:**
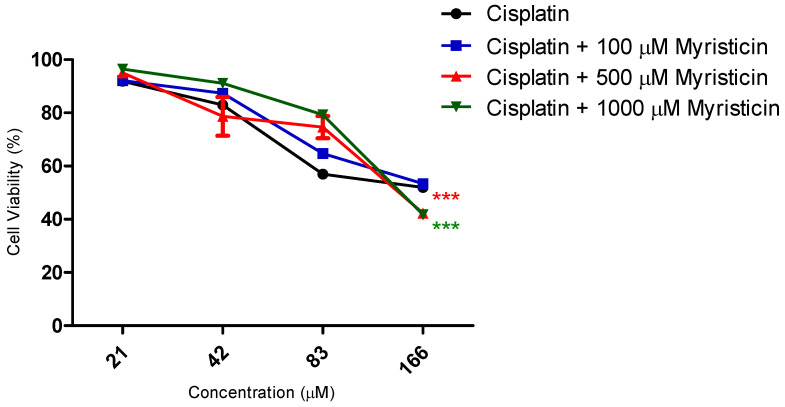
Cytotoxic activity of the compound myristicin in association with the chemotherapy drug cisplatin. Assay performed with the NCI/ADR-RES tumor line, relating the percentage of cell viability versus concentration of cisplatin, after 48 h of incubation.

**Figure 4:**
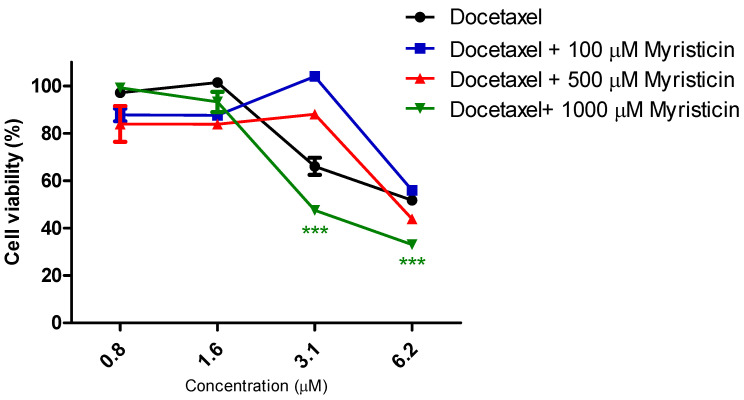
Cytotoxic activity of the compound myristicin in association with the chemotherapy drug docetaxel. Assay performed with the NCI/ADR-RES tumor line, relating the percentage of cell viability versus concentration of docetaxel, after 48 h of incubation.

Although it did not show cytotoxic activity in isolation (Figure 2) in the resistant tumor line, myristicin corroborated the potentiation of the effect of chemotherapeutics (Figure 3 and Figure 4), demonstrated by the reduction of IC50 values found for them when they were associated with myristicin (Table 1 and Table 2). With regard to cisplatin, this reduction was significant for the highest concentration of myristicin associated with the chemotherapy agent (1 mM). For docetaxel, it was observed that the two highest concentrations (500 µM and 1 mM) reduced the IC50 value. At the concentration of 1 mM, the CRI obtained for cisplatin was 1.49, which means that myristicin potentiated the effect of this chemotherapy agent by 1.49 times and reduced the IC50 value by 32.88%. At the same concentration, the CRI obtained for docetaxel was 4.08, demonstrating that myristicin potentiated the effect of this drug by 4.08 times and reduced the IC50 value by 75.46%. These CRI values above 1 indicate that there was synergism between myristicin and the chemotherapeutic agents used. At the lowest concentration, myristicin presented CRI < 1 for cisplatin and docetaxel, but this apparent worsening of effect was not statistically significant. Figure 3 and Figure 4 show that the effect of association of 100 µM myristicin and chemotherapeutics was not different from the effect of the chemotherapeutics alone.

**Figure 5:**
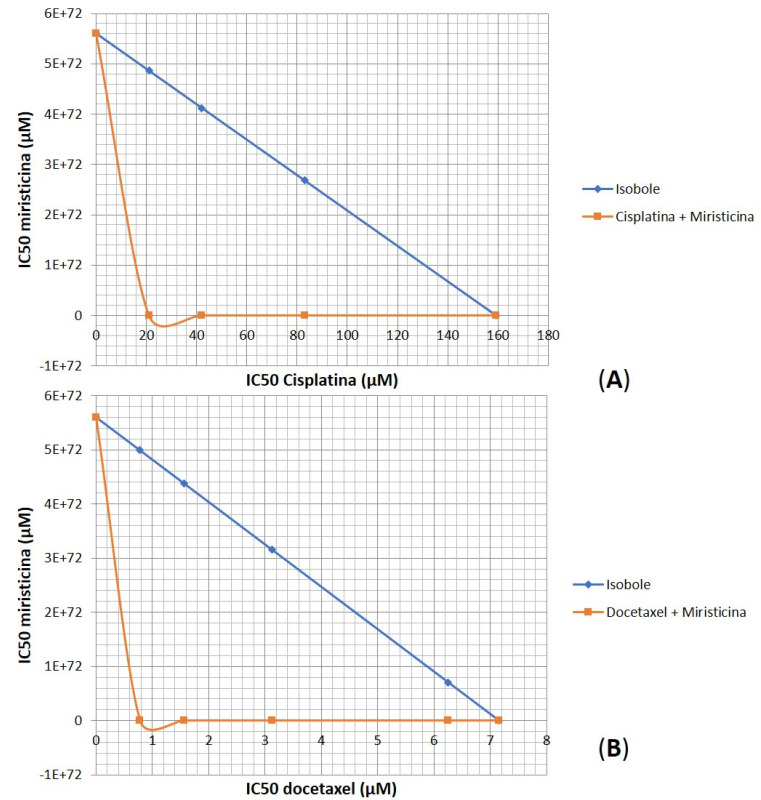
Isobolograms representing the synergistic interaction between myristicin and the chemotherapeutic agents cisplatin (**A**) and docetaxel (**B**) in the NCI/ADR-RES multidrug-resistant ovarian line after 48 h of incubation.

Considering the relevant IC50 and CRI values obtained for the highest concentration evaluated, there was an urgent need to prove whether the effect of the association between myristicin and chemotherapeutics was synergistic. The analysis of isobolograms was used to evaluate the interactions between myristicin and chemotherapeutics (Figure 5). The isobol represented in blue in the graph indicates the predictive additive effect of the association between myristicin and chemotherapeutics. The curve obtained below this isobol and represented in red indicates that the real effect of this association was synergistic, which means the resulting action is greater than the simple sum of the isolated effects of each one of them.

### 2.2. Molecular Docking

**Figure 6:**
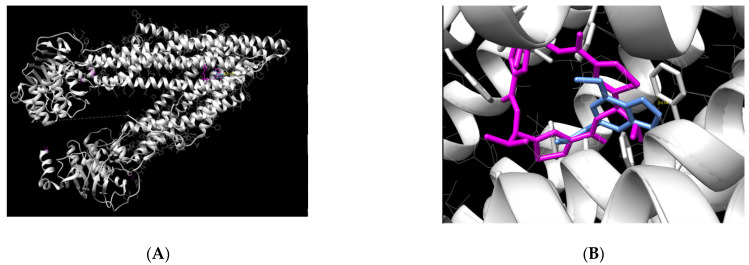
Graphic representation of the binding mode of P-glycoprotein and myristicin (**A**). Detail of the protein showing its sites occupied by myristicin and the natural ligand (**B**). The ligands are represented by pink (natural ligand) and blue (myristicin); P-gp is represented bygray.

Molecular docking was performed to evaluate the binding capacity of myristicin to P-gp. The result demonstrated that the myristicin molecule is able to bind at the center of the P-gp action site (Figure 6). This binding occurred in a very similar way to its natural ligand using a binding energy of −6.81 kcal/mol, which is considered an adequate value for a stable binding.

The chemical name of the ligand is (4R,11R,18R)-4,11,18-tri(propan-2-yl)-6,13,20-triselena-3,10,17,22,23,24-hexaazatetracyclo[17.2.1.1~5,8~.1~12,15~]tetracosa-1(21),5(24),7,12(23),14,19(22)-hexaene-2,9,16-trione.). Through the UCSF Chimera program, it was possible to verify which myristicin residues are able to bind to P-gp amino acids. The strongest binding found was between the C11 ligand of myristicin and the amino acid Phe 728 of P-gp, with a distance of 5.01 Å. This distance is very similar to that of the natural P-gp ligand with this amino acid. In addition, because it is a connection between two rings, it is considered very strong.

### 2.3. In Silico PK

According to the study led by Lipinski, there are five aspects of the molecule that must be considered to predict its permeability through biological membranes and, therefore, assess its pharmacokinetics and bioavailability [9]. Table 3 shows data obtained from myristicin and the values recommended by the rule.

The logP parameter is also called the oil–water partition coefficient. The ideal parameter for a drug is to have a logP lower than 5. Therefore, the logP of myristicin indicates that it has a good affinity for oil and water, favoring permeation through plasma membranes and reflecting good gastrointestinal absorption. In addition, drugs must have a molecular weight of less than 500 g/mol to have good permeability, as a very bulky molecule is more difficult to transport. Myristicin has a suitable molecular weight. Interactions with hydrogen occur mainly in aqueous media. The more ionic bonds the molecule makes with water, the more unfavorable its transport through membranes (which have the lipid component). According to Lipinski, the molecule must have less than 5 donors and less than 10 hydrogen acceptors to be a good drug; myristicin presented 0 and 3, respectively, fitting these parameters. The number of rotatable bonds can influence the bioavailability and binding potency, as the molecule must assume a fixed conformation to pass through membranes. The fewer rotatable bonds, the stiffer the molecule. A good drug candidate should have less than 10. Myristicin has only three, which is within the proper parameters.

## 3. Discussion

Cancer remains among the most serious diseases, although its treatment options are well established. There are many types of cancer treatment, depending on the type and at what stage it is. Chemotherapy is often used to treat cancer and well-designed drug delivery regimens have been effective in treating cancer and causing fewer adverse effects [10]. In some cases, the treatment plan may use a combination of methods to have maximum therapeutic effectiveness [11].

Platinum-derived chemotherapeutics are used as the main treatment for ovarian cancer despite their serious adverse effects and development of resistance. In clinical trials, cisplatin is often selected because of its strong antitumor activity, but its adverse effects include renal toxicity, nausea and vomiting. Therefore, to avoid renal toxicity, urine volumes must be monitored, and large-dose infusion is mandatory in cisplatin-based chemotherapy. The molecular mechanism of cisplatin-induced apoptosis involves activation of tumor protein 53 (p53), phosphorylation of the activator protein component (AP-1) leading to cell cycle arrest through stimulation of p21 and downregulation of cyclins and cyclin-dependent kinases [12].

Docetaxel is a semi-synthetic taxane that inhibits microtubule depolymerization, arresting cells in the G2/M phase of the cell cycle and induces bcl-2 phosphorylation, thus promoting a cascade of events that ultimately leads to apoptotic cell death [13]. It is approved for the treatment of breast and lung cancer and is indicated for the treatment of metastatic ovarian carcinoma after failure of first-line chemotherapy. Docetaxel is an important anticancer drug that can induce hypersensitivity reactions, such as blood hypereosinophilia, leading to deleterious treatment interruptions. Blood hypereosinophilia can be a potentially lethal biological sign of late visceral hypersensitivity reactions [14].

Although most ovarian tumors initially respond to chemotherapy, tumors often arise as a result of the expansion of clones with innate or acquired resistance, which later develop into recurrent tumors [15]. Certain tumor cells acquire a chemotherapy-resistant phenotype, resulting in treatment difficulties [10]. Statistical data show that more than 90% of cancer patient mortality is attributed to drug resistance [16]. Multidrug resistance (MDR) is a prominent mechanism of resistance to clinically approved therapies in ovarian cancer patients, and P-gp is one of the best-studied proteins involved in MDR.

Historically, natural products have played a key role in drug discovery, especially for cancer and infectious diseases [17]. The data show that there are still few drug discovery programs based on natural products in pharmaceutical companies, although they are a promising source of new drugs. Even so, drugs produced from natural substances are numerous, as they represent about 70% of all drugs approved for therapeutic use in the last four decades. Natural compounds have been one of the main sources of drug production since the beginning of time, giving rise to drugs of different therapeutic classes. Therefore, since the main source of new medicines are natural products, it is necessary to carry out research to discover new treatments from sources that are little explored [18].

The interest in investigating the in vitro effects of the association between myristicin and chemotherapeutics in an ovarian tumor cell line resistant to multiple drugs arose from the results obtained and published by our group for the apiole molecule, which differs from myristicin only by the presence of one more methoxy group [8]. It is a phenylpropanoid found mainly in parsley (Apiaceae) and in species of the families Lauraceae and Piperaceae. Apiole alone has no significant cytostatic effect on cell lines of resistant tumors. However, its association with the chemotherapy drugs vincristine and doxorubicin presented a synergistic effect, and this mechanism would be related to the affinity of the molecule to the active site of P-gp, antagonizing its action to promote drug efflux into the extracellular environment [8].

It has already been described in the literature that a higher number of methoxy groups that accept hydrogen bonds at the terminal of phenolic rings is favorable for the inhibitory activity of P-gp. Studies suggest that this chemical group acts as an additional acceptor of hydrogen bonding, implying a greater affinity for the active site of P-gp and resulting in potent inhibition of this protein [19]. However, although myristicin contains one methoxy group less than apiole, the binding energy observed in the in silico study was more negative than that demonstrated by apiole, suggesting that the binding is even more stable.

The molecular docking results indicated that myristicin is able to bind and block P-gp. This mechanism may be related to the improvement in the efficacy of chemotherapeutic agents, as it would allow a reduction in the dose of drugs and limit their cytotoxicity.

Another important tool for the study of drug interactions has been the construction of isobologram-type graphs. These are constructed from the IC50 values by generating the response curves and are used to prove synergism between molecules. The probability of interaction occurring after combining drugs increases, which can affect effectiveness and cause safety issues. If the combination of clinical drugs is much more effective than the sum of their individual effects, synergism between the drugs results, but if the therapeutic effect is weakened, the effect is one of antagonism. Synergy means that two or more components are mixed together and the effect is greater than the sum of the effects of the individual components when applied alone, thus producing the effect “1 + 1 > 2”. Within the scope of isobolographic analysis, antagonism occurs when the IC50 is greater than the expected concentrations of drugs A and B that are necessary to produce the target effect [20].

The results obtained in this study show that myristicin at a concentration of 1 mM potentiated the cytotoxic effects of chemotherapeutic agents, as evidenced by the decrease in IC50 values obtained and CRI values greater than 1. This effect is synergistic, since the effect of the association is greater than the sum of the effects of individual components when applied alone. It is believed that this potentiation of chemotherapeutic effects is primarily due to the blockage of the MDR-related efflux pump. Clinical co-administration of drugs that inhibit the efflux promoted by transmembrane proteins in combination with anticancer drugs is considered a treatment modality to overcome MDR in anticancer therapy [21]. Our hypothesis for myristicin action is that, by blocking the extrusion of chemotherapeutic agents, it allows these agents to freely enter cells and perform their functions, stopping the cell cycle.

Considering the potential clinical use of myristicin in association with chemotherapeutic drugs, we evaluated in silico some parameters for druglikeness according Lipinski’s criteria. For a molecule to be considered a good candidate for a drug, it must present at least four values within the recommended parameters [10]. Through the computational model used, it was possible to verify that myristicin fits in five of these parameters, with the exception of TPSA. Therefore, this study indicates that myristicin has adequate pharmacokinetics to become a drug, as it has good permeability through membranes and consequent bioavailability.

## 4. Material and Methods

### 4.1. Compound

The compound myristicin was purchased at Sigma-Aldrich (Burlington, MA, USA) using the code 09237.

### 4.2. Cell Culture

The resistant ovarian tumor cell line (NCI/ADR) was obtained from the National Cancer Institute at Frederick MA-USA. Stock cultures were grown in complete medium: RPMI 1640 medium (Sigma-Aldrich, Burlington, MA, USA) supplemented with 5% fetal bovine serum (LGC Biotecnologia, Cotia, SP, Brazil) and 1% penicillin:streptomycin (LGC Biotecnologia, Cotia, SP, Brazil) mixture (1000 U·mL^−1^:1000 µg·mL^−1^) at 37 °C with 5% CO_2_.

### 4.3. Cytotoxicity Assay of Myristicin and the Chemotherapeutic Agents Cisplatin and Docetaxel

For this test, the colorimetric method 3-(4,5-dimethylthiazol-2-yl)2,5-diphenyl tetrazolium bromide (MTT, Sigma-Aldrich, Burlington, MA, USA) was used, which indirectly evaluates the cell viability by the mitochondrial enzymatic activity of living cells. Briefly, 5000 cells were inoculated in 100 μL of complete medium in each well of the 96 wells, which was incubated for 24 hours at 37 °C in a 5% CO_2_ atmosphere and a humid environment. After 24 hours, myristicin was diluted in DMSO (Synth, Diadema, SP, Brazil) stock solution at a concentration of 0.1 g/mL, (100 μL/well) in triplicate, and, subsequently, the plate was incubated for 48 h at 37 °C in atmosphere of 5% CO_2_ and humid environment. As a positive control, the chemotherapeutic agents, cisplatin (C-platin, Blau Farmaceutica, Cotia, SP, Brazil) at concentrations from 10 to 333 μM and docetaxel (Eurofarma, São Paulo, SP, Brazil) at concentrations from 0.8 to 25 μM (100 μL/compartment), were tested in triplicate. These concentrations were based on previous studies conducted by the research group. After 48 h of treatment, the treated cells were then stained with MTT. After 4 h (incubation period), the dye was solubilized with DMSO, and the absorbance data were analyzed in a microplate reader (Promega) and compiled in the elaboration of graphs relating the percentage of cell growth with the concentration of the sample. Through the Origin^®^ software, the linear regression of the curves obtained with the averages of the percentage of viable cells in comparison to the DMSO negative control and the IC50 was calculated (concentration that reduces 50% of the cell viability). This parameter is used to determine the cytotoxic potency of the sample.

### 4.4. Association Assay between Myristicin and Chemotherapeutic Agents (Cisplatin and Docetaxel)

After obtaining the IC50 values of myristicin and the chemotherapeutic agents docetaxel and cisplatin, the cells received joint treatments with myristicin–docetaxel and myristicin–cisplatin, respectively, during 48 h of incubation and, at the end of this treatment time, the cells were stained with MTT and absorbance data were analyzed and compiled into graphs relating the percentage of cell growth to the concentration of the sample, as already described.

### 4.5. Molecular Docking

The myristicin molecule was designed using the OpenBabel tool, considering 3D parameters and pH 7. The result was converted to mol.2 format for use in docking. The target protein, P-glycoprotein, was selected from the RCSB PDB (Protein Data Bank), access code 3G60, chain A. Finally, the protein and the ligand were submitted to the SwissDock platform, which was able to predict the binding energy and position of myristicin in relation to P-gp.

The UCSF Chimera 1.15 program was used for data processing in order to verify the positioning of the molecule in P-gp and the overlap with the ligand. This program was also used to calculate the binding in the P-gp amino acids and the distance between the ligand and the protein.

### 4.6. In Silico PK

Pharmacokinetic parameters were calculated based on the molecular structure of myristicin and compared to the criteria established by Lipinski. The Molinspiration platform [22] was used to design the molecule and then obtain parameters related to physicochemical properties, solubility, lipophilicity.

## 5. Conclusions

Myristicin alone has no cytotoxic effect toward the resistant ovarian tumor lineage NCI/ADR-RES, but it promotes a synergistic effect when associated with the chemotherapy drugs cisplatin and docetaxel, reducing the chemotherapeutic concentration necessary to cause a 50% decrease in cell viability.

Considering the great obstacle in the chemotherapeutic treatment of cancer that is MDR acquired by tumor cells, investigating effective targets to circumvent this resistance remains an important challenge that needs to be solved. Therefore, this study encourages the continuation of the investigation of myristicin as a potential compound for the reversal of MDR.

## Figures and Tables

**Table 1:** Cytotoxic activity expressed as IC50 (µM) of myristicin and cisplatin and their association in the NCI/ADR-RES ^a^ lineage after 48 h of exposure.

	Myristicin	Cisplatin	Myristicin 1 mM + Cisplatin	Myristicin 500 µM + Cisplatin	Myristicin 100 µM + Cisplatin
IC50 ^b^	>1000	215.60 ± 6.36	144.70 ± 2.44 **	176.15 ± 21.61	259.86 ± 5.82
CRI ^c^	n.a.	n.a.	1.49	1.22	0.67

^a^ NCI/ADR-RES: multidrug-resistant ovarian adenocarcinoma cell line; ^b^ IC50: sample concentration required (µM) to inhibit 50% of cell viability and calculated by non-linear regression analysis using ORIGIN 7.5^®^ (OriginLab Corporation, Northampton, MA, USA); ^c^ CRI: concentration reduction index calculated as IC50 cisplatin/IC50 cisplatin + myristicin; n.a.: not applied. Statistical analysis by one-way ANOVA followed by Tukey’s test (** *p* < 0.01 related to cisplatin). The experiments were performed in biological triplicate and experimental duplicate. Concentration tested: 1.6–1000 µM (myristicin) and 5–332 µM (cisplatin).

**Table 2:** Cytotoxic activity expressed as IC50 (µM) of myristicin and docetaxel and their association in the NCI/ADR-RES ^a^ lineage after 48 h of exposure.

	Myristicin	Docetaxel	Myristicin 1 mM + Docetaxel	Myristicin 500 µM + Docetaxel	Myristicin 100 µM + Docetaxel
IC50 ^b^	>1000	15.04 ± 1.36	3.69 ± 0.00 **	10.90 ± 0.04 *	22.23 ± 2.78
CRI ^c^	n.a.	n.a.	4.08	1.38	0.83

^a^ NCI/ADR-RES: multidrug-resistant ovarian adenocarcinoma cell line; ^b^ IC50: sample concentration required (µM) to inhibit 50% of cell viability and calculated by non-linear regression analysis using ORIGIN 7.5^®^ (OriginLab Corporation, Northampton, MA, USA); ^c^ CRI: concentration reduction index calculated as IC50 docetaxel/IC50 docetaxel + myristicin; n.a.: not applied. Statistical analysis by one-way ANOVA followed by Tukey’s test (** *p* < 0.01; * *p* < 0.05, related to docetaxel). The experiments were performed in biological triplicate and experimental duplicate. Concentration tested: 1.6–1000 µM (myristicin) and 0.4–25 µM (docetaxel).

**Table 3:** Predicted pharmacokinetic parameters for myristicin compared to Lipinski’s criteria.

Parameter	Criteria	Myristicin
LogP	2 a 5	2.44
Molecular Weight	<500 g/mol	192.21 g/mol
Hydrogen-bond acceptors (HBAs)	<10	3
Hydrogen-bond donors (HBDs)	<5	0
Number of rotatable bonds	<10	3
Topological polar surface area (TPSA)	40 a 100 Â²	27.70

## Data Availability

Data is contained within the article.
